# Trends, differences, and future projections of lung cancer attributable to secondhand smoke across 204 countries and territories from 1990 to 2036

**DOI:** 10.18332/tid/202228

**Published:** 2025-03-28

**Authors:** Yulong Yu, Aifeina Aili, Bili Wu, Weiheng Zhao, Mu Yang, Xianglin Yuan

**Affiliations:** 1 Department of Oncology, Tongji Hospital, Tongji Medical College, Huazhong University of Science and Technology, Wuhan, China

**Keywords:** lung cancer, secondhand smoke, disease burden, prevention, health inequality

## Abstract

**INTRODUCTION:**

Secondhand smoke (SHS) has long been identified as a significant risk factor for lung cancer, yet the precise magnitude of its contribution to the global lung cancer burden remains unclear. Our study aims to elucidate the harms associated with lung cancer caused by secondhand smoke while emphasizing the importance of avoiding SHS.

**METHODS:**

The annual deaths and disability-adjusted life years (DALYs) data were obtained from the Global Burden of Disease Study (GBD) 2021 for this secondary dataset analysis. Trends in mortality and DALYs were evaluated, along with correlations with the sociodemographic index (SDI). Projections from 2021 to 2036 utilized a Bayesian age-period-cohort model.

**RESULTS:**

In 1990, SHS-related lung cancer was responsible for approximately 0.058 million deaths and 1.599 million DALYs, globally. By 2021, these numbers had increased to approximately 0.098 million deaths and 2.356 million DALYs worldwide. Between 1990 and 2021, SHS-related lung cumulatively caused 2.428 million deaths and 62.785 million DALYs. From 1990 to 2021, deaths and DALYs increased significantly, while age-standardized mortality rates (ASMR) and age-standardized DALY rates (ASDR) decreased. Specifically, ASMR decreased from 1.45 to 1.14, and ASDR dropped from 38.40 to 26.93. The high-middle SDI region bore the largest burden, accounting for nearly 40% of global deaths and DALYs. East Asia held the highest burden of lung cancer attributable to secondhand smoke in 2021, while Oceania had the lowest burden. Projections suggest that male ASMR will decline to 1.18 by 2036, while female ASMR is expected to rise to 0.91 by 2029 before decreasing to 0.89 by 2036.

**CONCLUSIONS:**

The considerable burden of lung cancer attributable to secondhand smoke underscores the urgent need for targeted public health interventions, particularly in high-risk demographics and regions. To mitigate disparities and enhance global health outcomes, it is crucial to prioritize the avoidance of SHS and the establishment of smoke-free environments.

## INTRODUCTION

Lung cancer remains a critical global health issue, representing the foremost cause of cancer-related mortality worldwide. In 2022, it was responsible for over 2.5 million new cases and 1.8 million deaths, thereby ranking first among all cancers in terms of both incidence and mortality^1^. In 2021, lung cancer ranked as the seventeenth leading cause of disability-adjusted life years (DALYs), accounting for 46.5 million DALYs^2^.

This burden of lung cancer is exacerbated by exposure to secondhand smoke (SHS), which affects millions of individuals, particularly non-smokers. SHS is a dangerous mixture of >7000 chemicals, of which hundreds are toxic and at least 70 have been identified as carcinogens^3,4^. Even short-term exposure to SHS is associated with adverse health outcomes, particularly affecting the respiratory and cardiovascular systems. Prolonged exposure is a known contributor to the development of lung cancer^5^. In fact, it is estimated that SHS increases lung cancer risk by approximately 30%^3^. Unfortunately, an estimated 37% of the global population is still exposed to smoke from burning tobacco products or exhaled by smokers, with women and children disproportionately affected compared to men^6^. Although awareness regarding the hazards of SHS is on the rise, the comprehensive scope of lung cancer cases attributable to SHS remains inadequately understood. While the Global Burden of Disease (GBD) study offers essential epidemiological insights, further analysis is necessary to elucidate the complexities of SHS-related lung cancer risk across different regions, ages, and sexes^7,8^. Previous studies of GBD have provided rough estimates of the contributions of various risk factors to the lung cancer burden^9^. However, these studies did not systematically analyze the impact of individual risk factors, making it difficult to assess the specific burden of lung cancer attributable to each factor. As a result, the significance of these risk factors may not have received adequate attention.

This study quantified the global burden of lung cancer attributable to SHS from 1990 to 2021 using data from the GBD study across 204 countries and territories, while also projecting trends for the next 15 years. By examining the association between SHS exposure and lung cancer incidence, this research provides critical insights that could help shape public health policies and strategies aimed at reducing SHS exposure. The findings are instrumental in guiding global efforts to lessen the burden of SHS-related lung cancer, ultimately improving health outcomes.

## METHODS

### Data source

This study is a secondary dataset analysis, utilizing data obtained from the Global Burden of Disease (GBD) database, specifically the GBD 2021 dataset. Lung cancer is defined by the development of tumors in the trachea, bronchi, or lungs. According to the International Classification of Diseases, 10th Revision (ICD-10), the corresponding codes for lung cancer include C33–C34.9, D02.1–D02.3, D14.2–D14.3, and D38.1^10^. We analyzed data related to lung cancer mortality and DALYs attributable to secondhand smoke exposure across 204 countries and territories from 1990 to 2021. The GBD database provides estimates of the burden of diseases, injuries, and risk factors globally, regionally, and nationally, standardized by demographic and epidemiological measures. The following is a brief description of GBD 2021:

1. Area: GBD 2021 covered 204 countries and territories, grouped into 21 regions and 7 super-regions. These regions were further classified based on the sociodemographic index (SDI), a composite measure reflecting a country’s per capita income, average education level, and total fertility rate. SDI values range from 0 (lowest) to 1 (highest), categorizing countries into five levels: low, low-middle, middle, high-middle, and high SDI.
2. Causes: GBD 2021 organized diseases and injuries into four hierarchical levels. Level 1 causes included communicable, maternal, neonatal, and nutritional diseases, along with non-communicable diseases (NCDs) and injuries. Level 2 causes encompassed neoplasms, cardiovascular diseases (CVD), chronic respiratory diseases (CRD), and others. Level 3 causes included ischemic heart disease (IHD), lower respiratory infections (LRIs), and breast cancer (BC), among others. At Level 4, more specific conditions such as ischemic stroke, diabetes mellitus type 2, and intracerebral hemorrhage were examined. In GBD 2021, multiple level 2 and level 3 causes were identified across both sexes.

### Study population and variables

The study included data from both male and female populations across all age groups. These data are subsequently integrated into a series of cause-of-death models to estimate lung cancer mortality, taking into account variables such as location, year, age, and sex^9^. The primary outcomes assessed were age-standardized mortality rates and age-standardized disability-adjusted life year rates attributable to secondhand smoke. Additional analyses were conducted based on sociodemographic index regions and sex-specific trends. The most accurate metric for evaluating disease burden is disability-adjusted life years, which integrate both years of life lost due to premature death (YLLs) and years lived with disability (YLDs). The sociodemographic index, a key tool in GBD analysis, assesses the relationship between disease burden and sociodemographic development^10^. The SDI ranges from 0 (lowest) to 100 (highest) and is based on three critical factors: total fertility rate under age 25 years (TFU25), mean education level of individuals aged ≥15 years (EDU15+), and lag-distributed per capita income^11^. Initially constructed for GBD 2015 using the human development index (HDI) methodology, the SDI assigns a 0 to 1 index value to each of these variables, with scales set by observed minima and maxima across the estimation period. In response to ongoing feedback and the evolving needs of GBD, the indicator has been refined with each GBD cycle. Based on SDI scores, 204 countries and territories were classified into five quintiles: low, low-middle, middle, high-middle, and high SDI^10^.

### Data extraction and management

Data were extracted using the Global Burden of Disease Results Tool, which provides comprehensive access to a wide array of health metrics. Key variables, including lung cancer mortality rates, DALYs, and attributions to specific risk factors such as secondhand smoke exposure, were downloaded for subsequent analysis. To ensure data integrity, the extracted datasets were meticulously cleaned and organized using Microsoft Excel and R software (version 4.4.1). The cleaning process involved identifying and rectifying any inconsistencies, including missing or anomalous values, following the stringent guidelines outlined in the GBD methodology. These guidelines include a systematic approach for handling gaps in data, such as imputation techniques or the exclusion of unreliable entries, to maintain the robustness of the final analysis. Additionally, descriptive statistics and exploratory analyses were performed to validate the quality of the data before proceeding with more detailed statistical assessments.

### Frontier analysis

We developed a data envelope analysis using the free disposal hull method to delineate non-linear frontiers for age-adjusted DALY rates based on SDI data from 1990 to 2021. To account for uncertainty, we generated 1000 bootstrapped samples by randomly sampling with replacement from all countries across all years. We then computed the mean DALYs at each SDI value from these bootstrapped samples. To produce a smoothed frontier, we applied LOESS regression with a local polynomial degree of 1 and a span of 0.2. Super-efficient countries were excluded to minimize the influence of outliers. To assess the relationship between age-standardized DALY rates and the frontier in 2021, we calculated the effective difference, defined as the absolute distance from the frontier using each country’s 2021 SDI and age-standardized DALY rate. Countries with DALY rates below the frontier were assigned a distance of zero.

### Statistical analysis

Descriptive analyses were performed to summarize the overall trends in lung cancer mortality and DALYs. ASMR and ASDR were calculated using the direct standardization method based on the global standard population. The slope index of inequality (SII) was utilized to quantify health disparities across socioeconomic groups. This metric measures the absolute difference in health outcomes between the lowest and highest socioeconomic groups, derived from a regression model that associates health outcomes with socioeconomic rank. A positive SII value indicates improved health outcomes with higher socioeconomic status, whereas a negative value suggests the opposite. The magnitude of the SII reflects the degree of inequality. Bayesian analysis was employed to estimate model parameters and their associated uncertainty. This approach integrates prior knowledge with observed data to generate posterior distributions, which are particularly advantageous for handling sparse data and complex models, such as projections of future mortality rates. Results are presented as posterior medians with 95% credible intervals (CrIs), providing a probabilistic range for the estimates. In this study, uncertainty intervals (UI) are used to express the range within which we believe the true value of a measurement (such as a death rate or disease burden) is likely to fall. These intervals account for various sources of uncertainty, such as variability in the data, limitations in the measurement methods, and assumptions made during the analysis a linear model was formulated to describe the relationship between the natural logarithm of the ratio and time, represented by the equation y = α + βx + ε, where x denotes the year and y signifies the ln(rate). The estimated annual percentage change (EAPC) was determined using the formula 100×(e^β^ - 1), accompanied by a 95% confidence interval (95% CI). Spearman correlation analysis was conducted to estimate the R indices and p-values for the association between age-standardized rates and the SDI. We fitted smooth splines using the locally weighted scatterplot smoothing method, which automatically determines the degree, number, and location of nodes (knots) on the basis of the data and the span parameter. The concentration curve is utilized to assess the relative disparity in the burden among countries by fitting the Lorenz concentration curve based on cumulative DALYs and cumulative population ranked by SDI. Statistical analyses were conducted using R software (version 4.4.1), and results were considered significant at a p<0.05.

## RESULTS

### Spatiotemporal distribution of lung cancer attributable to secondhand smoke

**Figure 1:**
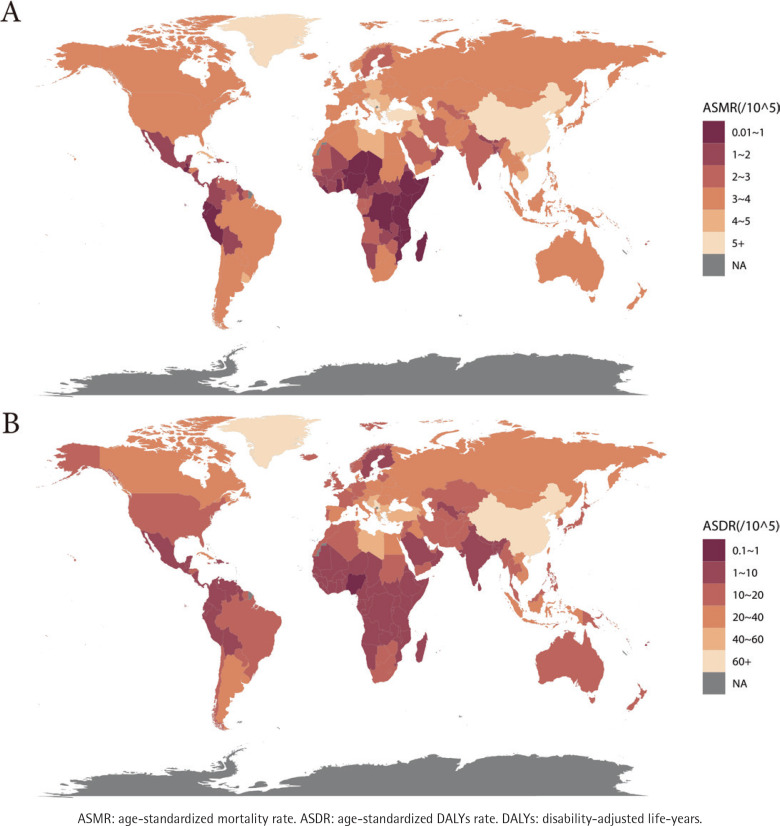
The global distribution of ASMR (A) and ASDR (B) of lung cancer attributable to secondhand smoke in 2021

In 2021, lung cancer attributable to secondhand smoke led to 98000 deaths and 2.356 million DALYs globally. Cumulatively, from 1990 to 2021, SHS-related lung cancer contributed to 2.428 million deaths and 62.785 million DALYs. Data indicate a significant increase in both deaths and DALYs attributable to secondhand smoke from 1990 to 2021. However, during the same period, the ASMR and ASDR exhibited a decline (Table 1, Figure 1). At the SDI regional level, the high-middle SDI region bore the greatest burden in 2021, with 39000 deaths and 936000 DALYs, representing almost 40% of the global impact (Supplementary file Figures S2A and S2B). This region also reported the highest ASMR and ASDR. From 1990 to 2021, all SDI regions saw reductions in ASMR and ASDR, with the most pronounced decline observed in the high SDI region, where ASMR (EAPC= -2.57; 95% CI: -2.67 – -2.46) and ASDR (EAPC= -2.93; 95% CI: -3.04 – -2.81) dropped significantly (Table 1, and Supplementary file Figure S1).

**Table1:** Global and regional burden of lung cancer attributable to secondhand smoke in 1990 and 2021, and EAPC of both ASMR and ASDR from 1990 to 2021

*Location*	*1990*				*2021*				*EAPC (1990–2021)*	
	*Deaths* *(95% UI)*	*ASMR per 100000* *(95% UI)*	*DALYs* *(95% UI)*	*ASDR per 100000* *(95% UI)*	*Deaths* *(95% UI)*	*ASMR per 100000* *(95% UI)*	*DALYs* *(95% UI)*	*ASDR per 100000* *(95% UI)*	*ASMR* *(95% CI)*	*ASDR* *(95% CI)*
**Global**	57618 (7083–107842)	1.45 (0.18–2.72)	1598871 (196922–2982788)	38.4 (4.72–71.68)	97911 (11955–184913)	1.14 (0.14–2.15)	2355866 (290211–4442996)	26.93 (3.32–50.83)	-0.88 (-0.94 – -0.82)	-1.25 (-1.31– -1.19)
**SDI region**										
**Low SDI**	374 (43–740)	0.17 (0.02–0.33)	10837 (1223–21437)	4.37 (0.5–8.65)	770 (94–1556)	0.15 (0.02–0.31)	22327 (2712–45068)	3.96 (0.48–8.01)	-0.43 (-0.52 – -0.35)	-0.49 (-0.58 – -0.4)
**Low-middle SDI**	2350 (311–4440)	0.39 (0.05–0.74)	67474 (8771–127412)	10.15 (1.33–19.21)	5478 (699–10459)	0.38 (0.05–0.73)	152292 (19444–291023)	9.9 (1.26–18.89)	-0.13 (-0.17 – -0.09)	-0.13 (-0.17 – -0.09)
**Middle SDI**	14192 (1799–25709)	1.41 (0.18–2.56)	401195 (50957–727467)	35.8 (4.54–64.87)	35511 (4497–67457)	1.36 (0.17–2.58)	862344 (110218–1626035)	31.13 (3.97–58.93)	-0.24 (-0.3 – -0.18)	-0.58 (-0.63 – -0.52)
**High-middle SDI**	21702 (2611–40729)	2.16 (0.26–4.04)	609304 (73612–1146504)	58.69 (7.08–110.37)	39124 (4613–73341)	1.96 (0.23–3.67)	936577 (111577–1736627)	47.2 (5.65–87.54)	-0.36 (-0.46 – -0.25)	-0.78 (-0.89 – -0.67)
**High SDI**	18917 (2323–36155)	1.74 (0.21–3.33)	507723 (62348–966857)	48.12 (5.91–91.54)	16945 (2216–32743)	0.82 (0.11–1.58)	380285 (49959–738240)	19.99 (2.63–38.76)	-2.57 (-2.67 – -2.46)	-2.93 (-3.04 – -2.81)
**GBD region**										
**East Asia**	21359 (2640–40336)	2.6 (0.32–4.9)	598410 (74708–1128102)	64.41 (7.99–121.28)	59196 (7267–111539)	2.75 (0.34–5.18)	1387975 (172611–2594496)	62.42 (7.79–116.4)	0.14 (0.01–0.26)	-0.19 (-0.29 – -0.08)
**Southeast Asia**	2122 (291–3992)	0.86 (0.12–1.63)	59389 (8258–112014)	21.65 (2.99–40.78)	5326 (696–10098)	0.83 (0.11–1.57)	142237 (18519–271986)	20.32 (2.66–38.74)	-0.33 (-0.42 – -0.24)	-0.41 (-0.5 – -0.32)
**Oceania**	18 (2–37)	0.67 (0.08–1.41)	492 (57–1030)	15.9 (1.83–33.37)	51 (7–113)	0.75 (0.1–1.66)	1411 (179–3141)	17.75 (2.26–39.22)	0.3 (0.23–0.38)	0.29 (0.21–0.37)
**Central Asia**	709 (92–1415)	1.45 (0.19–2.9)	21351 (2754–42678)	41.95 (5.4–83.79)	600 (78–1192)	0.73 (0.09–1.44)	16793 (2175–33398)	18.75 (2.44–37.2)	-1.8 (-1.95 – -1.65)	-2.24 (-2.36 – -2.12)
**Central Europe**	3808 (480–7293)	2.5 (0.32–4.79)	110561 (13890–211809)	72.72 (9.13–139.01)	3494 (438–6729)	1.61 (0.2–3.1)	86225 (10669–165607)	42.31 (5.22–81.04)	-1.46 (-1.64 – -1.27)	-1.8 (-2.01 – -1.59)
**Eastern Europe**	4323 (561–8351)	1.5 (0.19–2.9)	125653 (16106–243121)	43.66 (5.59–84.28)	2827 (354–5554)	0.8 (0.1–1.58)	75436 (9497–147003)	22.23 (2.83–43.42)	-2.1 (-2.26 – -1.93)	-2.3 (-2.47 – -2.13)
**High-income Asia Pacific**	2639 (344–5051)	1.32 (0.17–2.53)	65961 (8573–125048)	32 (4.16–60.71)	3440 (436–6957)	0.68 (0.09–1.4)	62673 (7816–128139)	14.83 (1.86–30.46)	-2.41 (-2.64 – -2.19)	-2.74 (-2.97 – -2.51)
**Australasia**	284 (30–604)	1.22 (0.13–2.61)	7576 (805–16022)	33.37 (3.56–70.55)	242 (26–526)	0.47 (0.05–1.01)	5814 (611–12570)	12.12 (1.27–26.13)	-3.04 (-3.14 – -2.95)	-3.2 (-3.28 – -3.12)
**Western Europe**	9027 (1054–17210)	1.65 (0.19–3.14)	244694 (28689–466150)	46.96 (5.53–89.32)	6227 (775–12307)	0.73 (0.09–1.43)	150799 (19175–295049)	19.46 (2.49–38.01)	-2.59 (-2.68 – -2.49)	-2.79 (-2.91 – -2.67)
**Southern Latin America**	692 (87–1406)	1.49 (0.19–3.03)	19432 (2494–39639)	41.45 (5.33–84.54)	614 (74–1314)	0.71 (0.08–1.52)	15436 (1845–33085)	18.3 (2.18–39.2)	-2.31 (-2.53 – -2.09)	-2.57 (-2.81 – -2.33)
**High-income North America**	7019 (857–13570)	2.09 (0.25–4.03)	188134 (22906–360573)	58.68 (7.12–111.97)	4568 (588–9059)	0.7 (0.09–1.38)	107413 (13713–211496)	17.36 (2.21–34.11)	-3.75 (-3.9 – -3.6)	-4.11 (-4.26 – -3.96)
**Caribbean**	269 (34–535)	1.07 (0.13–2.13)	6280 (799–12386)	23.99 (3.05–47.31)	357 (38–727)	0.66 (0.07–1.34)	8086 (863–16491)	14.96 (1.6–30.52)	-1.56 (-1.73 – -1.39)	-1.59 (-1.78 – -1.4)
**Andean Latin America**	48 (6–91)	0.24 (0.03–0.45)	1323 (168–2503)	6.06 (0.77–11.48)	67 (8–132)	0.11 (0.01–0.22)	1726 (201–3400)	2.84 (0.33–5.59)	-2.9 (-3.18 – -2.63)	-3.02 (-3.31 – -2.72)
**Central Latin America**	338 (42–634)	0.43 (0.05–0.8)	8867 (1096–16588)	10.18 (1.26–19.06)	455 (55–866)	0.18 (0.02–0.35)	11230 (1346–21277)	4.39 (0.53–8.31)	-3 (-3.12 – -2.89)	-3.02 (-3.14 – -2.9)
**Tropical Latin America**	916 (112–1758)	1.02 (0.12–1.96)	25378 (3109–48617)	26.02 (3.18–49.85)	1368 (161–2745)	0.53 (0.06–1.07)	34368 (4093–69074)	13.08 (1.56–26.27)	-2.3 (-2.4 – -2.19)	-2.44 (-2.53 – -2.34)
**North Africa and Middle East**	2151 (237–4062)	1.31 (0.15–2.46)	60176 (6579–114545)	33.1 (3.63–62.82)	4534 (526–8819)	1.04 (0.12–2.03)	120069 (13861–232534)	24.83 (2.88–48.46)	-0.78 (-0.89 – -0.66)	-0.99 (-1.09 – -0.89)
**South Asia**	1481 (186–2775)	0.26 (0.03–0.5)	42798 (5403–79876)	6.78 (0.85–12.68)	3869 (526–7497)	0.26 (0.04–0.51)	108253 (14692–210038)	6.85 (0.93–13.28)	-0.42 (-0.6 – -0.24)	-0.35 (-0.52 – -0.17)
**Central Sub-Saharan Africa**	38 (5–79)	0.16 (0.02–0.34)	1168 (143–2457)	4.47 (0.54–9.33)	84 (10–180)	0.14 (0.02–0.31)	2671 (297–5691)	3.99 (0.45–8.51)	-0.44 (-0.75 – -0.13)	-0.42 (-0.73 – -0.11)
**Eastern Sub-Saharan Africa**	91 (11–179)	0.12 (0.01–0.23)	2737 (336–5391)	3.24 (0.4–6.4)	142 (18–272)	0.08 (0.01–0.16)	4306 (533–8218)	2.21 (0.27–4.22)	-1.36 (-1.45 – -1.26)	-1.48 (-1.59 – -1.38)
**Southern Sub-Saharan Africa**	223 (27–440)	0.81 (0.1–1.6)	6711 (821–13291)	22.55 (2.75–44.64)	307 (40–584)	0.52 (0.07–0.99)	8950 (1160–17074)	14.07 (1.82–26.85)	-1.48 (-1.7 – -1.27)	-1.56 (-1.78 – -1.35)
**Western Sub-Saharan Africa**	64 (8–125)	0.07 (0.01–0.14)	1781 (227–3460)	1.89 (0.24–3.68)	142 (18–278)	0.07 (0.01–0.15)	3995 (492–7784)	1.85 (0.23–3.61)	-0.05 (-0.13–0.03)	-0.15 (-0.23 – -0.06)

ASMR: age-standardized mortality rate. ASDR: age-standardized DALYs rate. DALYs: disability-adjusted life-years. EAPC: estimated annual percentage change. SDI: sociodemographic index. UI: uncertainty interval. CI: confidential interval.

Regarding GBD regions, East Asia held the highest burden in 2021, contributing to nearly 60% of global secondhand smoke-related lung cancer deaths and DALYs, with the highest ASMR (2.75; 95% UI: 0.34–5.18) and ASDR (62.42; 95% UI: 7.79–116.40) (Supplementary file Figures S2C and S2D). Between 1990 and 2021, regions like the high-income Asia-Pacific, Eastern Europe, Southern Latin America, Australia, Western Europe, high-income North America, Andean Latin America, Central Latin America, and Tropical Latin America showed the fastest declines in ASMR and ASDR, with EAPCs exceeding^2^. High-income North America exhibited the steepest declines, with ASDR having an EAPC of -4.11 (95% CI: -4.26 – -3.96) and ASMR having an EAPC of -3.75 (95% CI: -3.9 – -3.6) (Table 1, and Supplementary file Figures S2E and S2F).

### Global lung cancer burden attributable to secondhand smoke by age and gender

**Figure 2:**
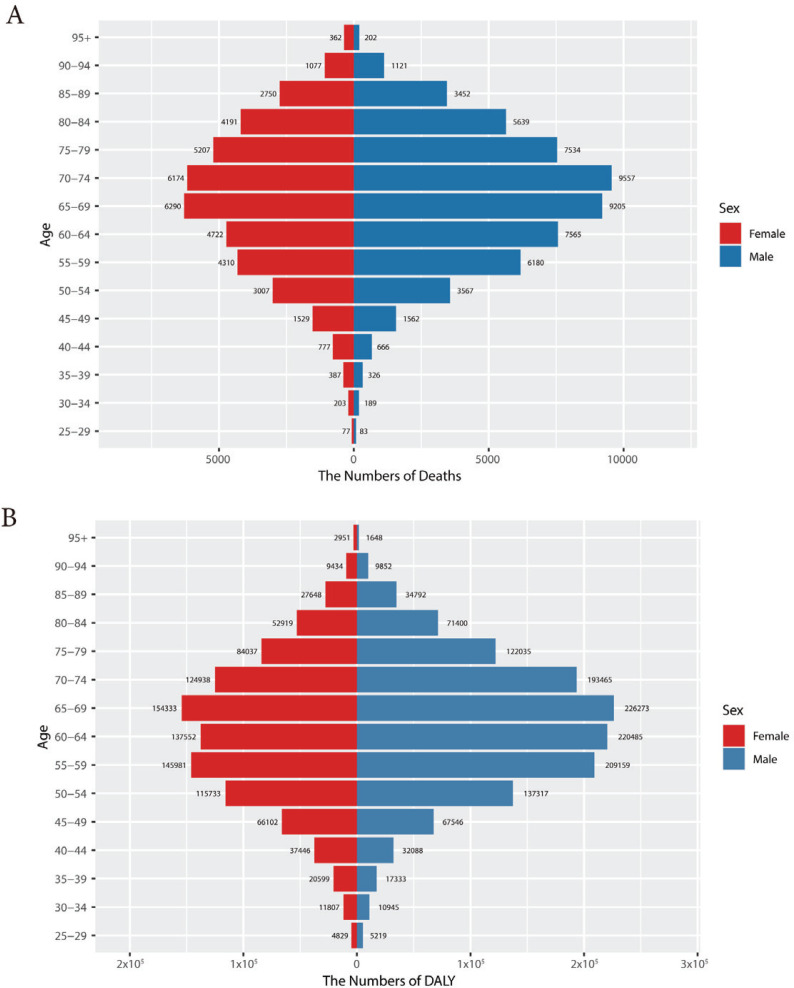
Global lung cancer burden attributable to secondhand smoke by age and gender. Number of deaths (A) and number of DALYs (B) of lung cancer attributable to secondhand smoke for different age groups in 2021

The global burden of lung cancer attributable to secondhand smoke in 2021 is depicted in Figure 2, with charts disaggregated by age group. The data highlights lung cancer mortality (Figure 2A), and DALYs (Figure 2B), providing a comprehensive analysis of the impact across different age cohorts. Lung cancer deaths due to secondhand smoke peaked in males aged 70–74 years and in females aged 65–69 years, with a gradual decline in deaths observed after these age groups. DALYs, for both males and females, reached their highest point in the age group of 65–69 years. Notably, in the age groups of 30–49 years and ≥95 years, the number of deaths and DALYs for females exceeds that of males. In all other age groups, males have higher rates than females. Age-specific mortality followed an inverted V-shaped distribution pattern, with a distinct mortality pattern related to secondhand smoke observed in 2021. Both male and female age groups demonstrated synchronous increases followed by subsequent declines in mortality rates. This finding underscores the significance of age as a contributing factor in the global cancer burden associated with secondhand smoke. Mortality rates peaked among individuals aged 85–89 years, with male mortality rates increasing up to this age range and then declining sharply. Similarly, female mortality rates rose before the age of 85–89 years but declined more gradually than those of males. Consequently, male mortality rates were significantly higher than those of females. The gender disparity in secondhand smoke-related lung cancer mortality was especially pronounced in individuals aged 85–89 years (Supplementary file Figure S3A). The age-specific DALYs burden of cancer mirrored the pattern of age-specific cancer mortality, peaking in the age group 75–79 years. Gender differences in DALYs attributable to secondhand smoke-related lung cancer were also prominent among individuals aged 70–89 years ( Supplementary file Figure S3B).

### The correlation between burden of SHS-related lung cancer and sociodemographic index

Figure 3 illustrates the correlation between the ASDR of lung cancer attributable to secondhand smoke and SDI at both regional and national levels, revealing a positive correlation in both contexts (Pearson’s R=0.5271, p<0.001). The positive R value indicates that ASDR tends to increase with higher SDI, suggesting a moderate association between socioeconomic development and the burden of lung cancer due to secondhand smoke. Regionally, the global ASDR surpassed the anticipated ASDR. Regions including East Asia, Central Europe, and high-income North America showed ASMR values exceeding expectations relative to their SDI from 1990 to 2021 (Supplementary file Figure S4A). Several countries, including Montenegro and North Macedonia, had observed rates significantly above expectations, while countries like Guyana and Peru had observed rates significantly below expectations, considering their SDI values (Supplementary file Figure S4B). A similar pattern was observed for ASMR (Supplementary file Figure S5).

The slope index of inequality (SII) for DALYs was 43.94 in 1990 and 33.15 in 2021, demonstrating a positive correlation between age-standardized DALYs rates and the SDI index (Supplementary file Figures S4C and S4D). The notable decrease indicates a narrowing of the disparity in age-standardized secondhand smoke-related lung cancer burden between high-SDI and low-SDI countries during this period. The concentration index for DALYs showed a decreasing trend from 1990 to 2021 (Supplementary file Figure S4E).

### Joinpoint regression analysis from 1990 to 2021 and the projected trends from 2021 to 2036

The joinpoint regression analysis of global ASMR and ASDR for secondhand smoke-related lung cancer was conducted from 1990 to 2021. We found that the ASMR for males showed a declining trend from 1990 to 2021 (APC= -2.14, 2004–2007; APC= -1.79, 2011–2019), while females exhibited an initial increase, followed by a decrease, and then a subsequent rise (APC=1.07; 1990–1995; APC= -1.11; 2000–2007; APC=0.25; 2015–2021) (Table 2, and Supplementary file Figures S6A and S6B). A similar pattern was observed in the ASDR (Supplementary file Figures S6C and S6D).

**Table 2:** Joinpoint regression analysis: global trends in age-standardized mortality and DALYs rates (per 100000 persons) among both sexes, males, and females, 1990–2021

*Gender*	*ASMR*			*ASDR*		
	*Period*	*APC (95% CI)*	*AAPC (95% CI)*	*Period*	*APC (95% CI)*	*AAPC (95% CI)*
**Both**	1990–1994	-0.0182 (-0.35–0.31)	-0.7758 (-0.93 – -0.63)	1990–1994	-0.2775 (-0.59–0.03)	-1.1431 (-1.2689 – -1.0172)
	1994–2004	-0.6034 (-0.70 – -0.51)		1994–2004	-0.9954 (-1.09 – -0.91)	
	2004–2007	-1.7756 (-2.79 – -0.75)		2004–2007	-1.9299 (-2.89 – -0.96)	
	2007–2010	-0.2466 (-1.28–0.80)		2007–2011	-0.9852 (-1.47 – -0.50)	
	2010–2018	-1.3554 (-1.49 – -1.22)		2011–2017	-1.8445 (-2.06 – -1.62)	
	2018–2021	-0.3278 (-0.85–0.20)		2017–2021	-0.8833 (-1.19 – -0.57)	
**Female**	1990–1995	1.0659 (0.82–1.31)	-0.323 (-0.44 – -0.20)	1990–1995	0.7706 (0.57–0.97)	-0.6503 (0.7496 – -0.5508)
	1995–2000	-0.4913 (-0.83 – -0.15)		1995–2000	-0.7941 (-1.07 – -0.51)	
	2000–2007	-1.1128 (-1.29 – -0.93)		2000–2007	-1.3242 (-1.47 – -1.18)	
	2007–2011	-0.1749 (-0.71–0.37)		2007–2011	-0.6753 (-1.12 – -0.23)	
	2011–2015	-1.4456 (-1.99 – -0.90)		2011–2015	-1.7415 (-2.18 – -1.30)	
	2015–2021	0.2487 (0.06–0.44)		2017–2021	-0.1676 (-0.32 – -0.01)	
**Male**	1990–1998	-0.9077 (-1.02 – -0.80)	-1.0889 (-1.22 – -0.96)	1990–1999	-1.2132 (-1.32 – -1.11)	-1.471 (-1.6252 – -1.3165)
	1998–2004	-0.3262 (-0.55 – -0.10)		1999–2004	-0.7721 (-1.13 – -0.41)	
	2004–2007	-2.1356 (-3.11 – -1.15)		2004–2007	-2.3815 (-3.49 – -1.26)	
	2007–2011	-0.5474 (-1.04 – -0.06)		2007–2011	-1.0854 (-1.6426 – -0.53)	
	2011–2019	-1.7887 (-1.92 – -1.66)		2011–2019	-2.1342 (-2.28 – -1.98)	
	2019–2021	-0.7847 (-1.78–0.22)		2017–2021	-1.1051 (-2.24–0.047)	

The joinpoint regression model’s calculation approach is to estimate the changing rule of illness rates using the least square method, avoiding the non-objectivity of typical trend analyses based on linear trends. AAPC: annual average percentage change. ASMR: age-standardized mortality rate. ASDR: age-standardized DALYs rate.

To understand the projected trends in secondhand smoke-related lung cancer ASMR and ASDR after 2021, we employed a Bayesian age-period-cohort model to predict ASMR and ASDR from 2021 to 2036 by gender. As shown in Supplementary file Figure S6E, the ASMR for males is projected to decline annually, dropping from 1.45 in 2021 to 1.18 in 2036. However, the forecast for females indicates an increase followed by a decline (Supplementary file Figure S6F), with ASMR rising from 0.89 in 2021 to 0.906 in 2029, before decreasing to 0.898 in 2036. These findings suggest a continuous downward trend in ASMR for males, while females are expected to see an initial increase followed by a decrease.

Both males and females are projected to experience annual declines in ASDR (Supplementary file Figures S6G and S6H), though the decrease is more pronounced in males. For males, the ASDR is forecast to drop from 33.00 in 2021 to 24.69 in 2036. For females, the ASDR is projected to decline from 21.87 in 2021 to 20.46 in 2036.

### Frontier analysis of age-standardized DALYs rates

From 1990 to 2021, the unrealized health gains in countries or regions with varying levels of development are shown in Supplementary file Figure S7A. Supplementary file Figure S7B illustrates the DALYs burden and efficiency gap across countries or regions with different sociodemographic development levels in 2021. As sociodemographic development advances, the efficiency gap generally increases, indicating that countries or regions with higher SDI have greater potential for improvement in disease burden reduction (Supplementary file Figure S7B).

## DISCUSSION

This extensive analysis of the global burden of lung cancer attributable to SHS from 1990 to 2021 provides critical insights into the spatiotemporal trends, epidemiological patterns, and health impacts across 204 countries and territories. The International Agency for Research on Cancer has classified SHS exposure, which can induce mutagenesis and promote lung carcinogenesis, as a group 1 carcinogen^11^. Despite heightened awareness and the implementation of tobacco control policies, SHS continues to pose a significant public health challenge, contributing to an estimated 98000 deaths and 2.356 million DALYs worldwide in 2021. From 1990 to 2021, the global burden of lung cancer attributable to SHS exposure has shown distinct temporal and regional trends. Although age-standardized mortality and DALY rates have generally declined, the absolute burden has increased, reflecting the influence of population aging and growth.

Our findings emphasize that, while overall age-standardized mortality and DALY rates have declined since 1990, these gains are not evenly distributed. High-middle SDI regions, such as East Asia and Central Europe, bear a disproportionately high burden of lung cancer attributable to SHS, with a peak in mortality and DALYs in 2021. Regions with high SDI, on the other hand, have experienced more pronounced declines in SHS-related mortality and DALY rates. Significant regional variations exist in the burden of lung cancer attributable to SHS, driven by differences in tobacco control policies, healthcare infrastructure, and socio-cultural factors^12,13^. High-income regions have generally achieved substantial reductions in SHS-related lung cancer due to strong tobacco control measures, including comprehensive bans on smoking in public places, rigorous enforcement of SHS exposure regulations, and widespread public health campaigns^14^. In our study, these regions have seen some of the steepest declines in ASMR and ASDR, with high-income North America showing the most pronounced reductions. In contrast, the high-middle SDI region, which includes several rapidly developing economies, has the highest absolute burden of SHS-related lung cancer. The high-middle SDI regions often lack stringent smoke-free policies and effective enforcement mechanisms, leading to high levels of SHS exposure in both public and private settings^15^. This disparity highlights the need for region-specific interventions that address local patterns of SHS exposure and target vulnerable populations, such as women and children, who are often disproportionately affected.

Joinpoint regression models revealed a consistent decline in ASMR among males from 1990 to 2021, while females showed an initial increase, followed by a decrease, and then a subsequent rise. Using a Bayesian age-period-cohort model, we projected ASMR and ASDR for the next 15 years. The projections indicate a continued decline in male ASMR from 2021 to 2036, whereas female ASMR is expected to increase over the first eight years before declining. The observed gender differences may be due, in part, to greater susceptibility to tobacco-related carcinogenesis among women^16^. Additionally, research indicates that females, particularly girls, report higher exposure to secondhand smoke in homes and other enclosed public spaces compared to males^17^, further exacerbating this public health concern. These findings highlight the need for increased focus on the burden of lung cancer attributable to secondhand smoke in females. It is essential to implement robust measures to prevent the increase of this burden in the female population.

The global health burden of SHS-related lung cancer calls for urgent policy interventions aimed at reducing SHS exposure and mitigating its adverse health effects^18^. The implementation of comprehensive smoke-free laws is a crucial step in curbing SHS exposure, as evidenced by the success of high-SDI regions in reducing SHS-related lung cancer incidence^19^. Policymakers should prioritize the adoption and enforcement of stringent smoke-free policies in high-middle SDI region, where SHS exposure remains high and tobacco control efforts are often hampered by limited resources and the influence of the tobacco industry^20^. Additionally, targeted public health campaigns are needed to raise awareness about the risks of SHS exposure, particularly in settings where smoking is culturally ingrained or where misconceptions about SHS persist^21^. Educational programs should focus on vulnerable populations, including non-smoking women, children, and the elderly, who are often involuntarily exposed to SHS in homes and workplaces. Such campaigns should be culturally tailored to resonate with local communities and empower individuals to advocate for smoke-free environments^22^.

Addressing the economic impact of SHS-related lung cancer is another critical policy consideration^23^. The financial burden associated with lung cancer treatment and the loss of productivity due to illness and premature death can strain healthcare systems, particularly in high-middle SDI region^24^. Investing in tobacco control measures, including SHS reduction, is not only a public health imperative but also a cost-effective strategy to alleviate the economic burden of tobacco-related diseases. Moreover, the impact of emerging tobacco products, such as e-cigarettes and heated tobacco products, on SHS exposure and lung cancer risk warrants further investigation. As these products gain popularity, particularly among youth and non-smokers, their potential to contribute to passive exposure and associated health risks must be closely monitored. Understanding these trends will be essential for developing effective, evidence-based tobacco control policies that address the evolving landscape of tobacco use and SHS exposure.

### Limitations

However, there are several limitations that should be considered in our study. First, the Joinpoint regression analysis and EAPC model have inherent limitations, including the oversimplification of complex temporal trends and potential bias from data-driven joinpoint selection. Projections using Bayesian age-period-cohort models assume that past trends will continue, which may not account for unexpected changes in tobacco use or public health interventions. Additionally, residual confounding from unmeasured factors like occupational exposures and genetic predispositions could skew estimates of lung cancer risk. In low- and middle-income regions, reliance on modeled estimates of SHS exposure may introduce inaccuracies due to scarce or low-quality data. Furthermore, variations in cancer registry coverage affect mortality and DALY estimates. Future research should focus on refining SHS exposure assessments and exploring interactions between SHS and other risk factors, such as occupational exposures and ambient air pollution, to provide a more comprehensive understanding of the determinants of lung cancer risk.

## CONCLUSIONS

Despite a decrease in ASMR and ASDR rates between 1990 and 2021, the global burden of lung cancer attributable to secondhand smoke has risen in terms of absolute deaths and DALYs. High-middle SDI regions, particularly East Asia, continue to shoulder the greatest burden, with significant gender disparities evident across all age groups. Regional variations also persist, with some countries displaying higher-than-expected rates relative to their socioeconomic development. Although high-income regions have achieved faster reductions in ASMR and ASDR, the overall inequality in the burden of secondhand smoke-related lung cancer remains a persistent challenge. The findings provide robust evidence to inform public health policies aimed at adopting comprehensive tobacco control measures and promoting the development of smoke-free environments, thereby strengthening efforts to prevent lung cancer at the regional level.

## Supplementary Material



## Data Availability

The datasets used and/or analyzed during the current study are available from the corresponding author upon reasonable request.

## References

[1] Bray F, Laversanne M, Sung H, et al. Global cancer statistics 2022: GLOBOCAN estimates of incidence and mortality worldwide for 36 cancers in 185 countries. CA Cancer J Clin. 2024;74(3):229-263. doi:10.3322/caac.21834 38572751

[2] GBD 2021 Diseases and Injuries Collaborators. Global incidence, prevalence, years lived with disability (YLDs), disability-adjusted life-years (DALYs), and healthy life expectancy (HALE) for 371 diseases and injuries in 204 countries and territories and 811 subnational locations, 1990-2021: a systematic analysis for the Global Burden of Disease Study 2021. Lancet. 2024;403(10440):2133-2161. doi:10.1016/S0140-6736(24)00757-8 38642570 PMC11122111

[3] Štěpánek L, Ševčíková J, Horáková D, Patel MS, Durďáková R. Public health burden of secondhand smoking: case reports of lung cancer and a literature review. Int J Environ Res Public Health. 2022;19(20):13152. doi:10.3390/ijerph192013152 36293731 PMC9603183

[4] Yousuf H, Hofstra M, Tijssen J, et al. Estimated worldwide mortality attributed to secondhand tobacco smoke exposure, 1990-2016. JAMA Netw Open. 2020;3(3):e201177. doi:10.1001/jamanetworkopen.2020.1177 32181828 PMC7078760

[5] Barnoya J, Glantz SA. Cardiovascular effects of secondhand smoke: nearly as large as smoking. Circulation. 2005;111(20):2684-2698. doi:10.1161/CIRCULATIONAHA.104.492215 15911719

[6] Mbulo L, Palipudi KM, Andes L, et al. Secondhand smoke exposure at home among one billion children in 21 countries: findings from the Global Adult Tobacco Survey (GATS). Tob Control. 2016;25(e2):e95-e100. doi:10.1136/tobaccocontrol-2015-052693 26869598 PMC5488799

[7] Oberg M, Jaakkola MS, Woodward A, Peruga A, Prüss-Ustün A. Worldwide burden of disease from exposure to second-hand smoke: a retrospective analysis of data from 192 countries. Lancet. 2011;377(9760):139-146. doi:10.1016/S0140-6736(10)61388-8 21112082

[8] Eng L, Su J, Qiu X, et al. Second-hand smoke as a predictor of smoking cessation among lung cancer survivors. J Clin Oncol. 2014;32(6):564-570. doi:10.1200/JCO.2013.50.9695 24419133

[9] Kuang Z, Wang J, Liu K, et al. Global, regional, and national burden of tracheal, bronchus, and lung cancer and its risk factors from 1990 to 2021: findings from the global burden of disease study 2021. EClinicalMedicine. 2024;75:102804. doi:10.1016/j.eclinm.2024.102804 39290907 PMC11406099

[10] GBD 2019 Diseases and Injuries Collaborators. Global burden of 369 diseases and injuries in 204 countries and territories, 1990-2019: a systematic analysis for the Global Burden of Disease Study 2019. Lancet. 2020;396(10258):1204-1222. doi:10.1016/S0140-6736(20)30925-9 33069326 PMC7567026

[11] Mochizuki A, Shiraishi K, Honda T, et al. Passive smoking-induced mutagenesis as a promoter of lung carcinogenesis. J Thorac Oncol. 2024;19(7):984-994. doi:10.1016/j.jtho.2024.02.006 38382595

[12] Carreras G, Lugo A, Gallus S, et al. Burden of disease attributable to second-hand smoke exposure: a systematic review. Prev Med. 2019;129:105833. doi:10.1016/j.ypmed.2019.105833 31505203

[13] Hatsukami DK, Carroll DM. Tobacco harm reduction: past history, current controversies and a proposed approach for the future. Prev Med. 2020;140:106099. doi:10.1016/j.ypmed.2020.106099 32335031 PMC7581601

[14] Zhai C, Hu D, Yu G, et al. Global, regional, and national deaths, disability-adjusted life years, years lived with disability, and years of life lost for the global disease burden attributable to second-hand smoke, 1990-2019: a systematic analysis for the Global Burden of Disease Study. Sci Total Environ. 2023;862:160677. doi:10.1016/j.scitotenv.2022.160677 36481152

[15] Leung J, Lim C, Sun T, et al. Preventable deaths attributable to second-hand smoke in Southeast Asia-analysis of the Global Burden of Disease Study 2019. Int J Public Health. 2024;69:1606446. doi:10.3389/ijph.2024.1606446 39027013 PMC11254616

[16] Mollerup S, Berge G, Baera R, et al. Sex differences in risk of lung cancer: expression of genes in the PAH bioactivation pathway in relation to smoking and bulky DNA adducts. Int J Cancer. 2006;119(4):741-744. doi:10.1002/ijc.21891 16557573

[17] Huai B, Chang KC, Filippidis FT. Inequalities in exposure to second-hand smoke among adolescent boys and girls in 122 countries: evidence from the Global Youth Tobacco Survey. Prev Med. 2024;189:108146. doi:10.1016/j.ypmed.2024.108146 39353471

[18] Besaratinia A, Pfeifer GP. Second-hand smoke and human lung cancer. Lancet Oncol. 2008;9(7):657-666. doi:10.1016/S1470-2045(08)70172-4 18598930 PMC2740739

[19] Wang M, Maimaitiming M, Zhao Y, Jin Y, Zheng ZJ. Global trends in deaths and disability-adjusted life years of diabetes attributable to second-hand smoke and the association with smoke-free policies. Public Health. 2024;228:18-27. doi:10.1016/j.puhe.2023.12.025 38246128

[20] Kim HJ, Fay MP, Feuer EJ, Midthune DN. Permutation tests for joinpoint regression with applications to cancer rates. Stat Med. 2000;19(3):335-351. doi:10.1002/(SICI)1097-0258(20000215)19:3<335::AID-SIM336>3.0.CO;2-Z 10649300

[21] Schoretsaniti S, Filippidis FT, Vardavas CI, et al. Prevalence and determinants of SHS exposure in public and private areas after the 2010 smoke-free legislation in Greece. Int J Environ Health Res. 2014;24(5):401-411. doi:10.1080/09603123.2013.835033 24044769

[22] Murakami K, Obara T, Ishikuro M, Ueno F, Noda A, Kuriyama S. Associations of education and income with secondhand smoke exposure among non-smoking pregnant women in Japan: the Tohoku Medical Megabank Project Birth and Three-Generation Cohort Study. Matern Child Health J. 2023;27(7):1238-1246. doi:10.1007/s10995-023-03648-x 36988795

[23] Shafer P. Impact of US smoke-free air laws on restaurant and bar employment, 1990-2015. Nicotine Tob Res. 2019;21(4):547-550. doi:10.1093/ntr/ntx280 29309684

[24] Ezeife DA, Morganstein BJ, Lau S, et al. Financial burden among patients with lung cancer in a publically funded health care system. Clin Lung Cancer. 2019;20(4):231-236. doi:10.1016/j.cllc.2018.12.010 30797721

