# Supplementary material for: Trends, differences, and future projections of lung cancer attributable to secondhand smoke across 204 countries and territories from 1990 to 2036
Source: Tob Induc Dis. 2025 Mar 28;23:10.18332/tid/202228. doi: 10.18332/tid/202228 (PMC11951970; doi:10.18332/tid/202228)
Supplement: Supplementary file 1 [file TID-23-43-s1.pdf]

Supplementary file

A

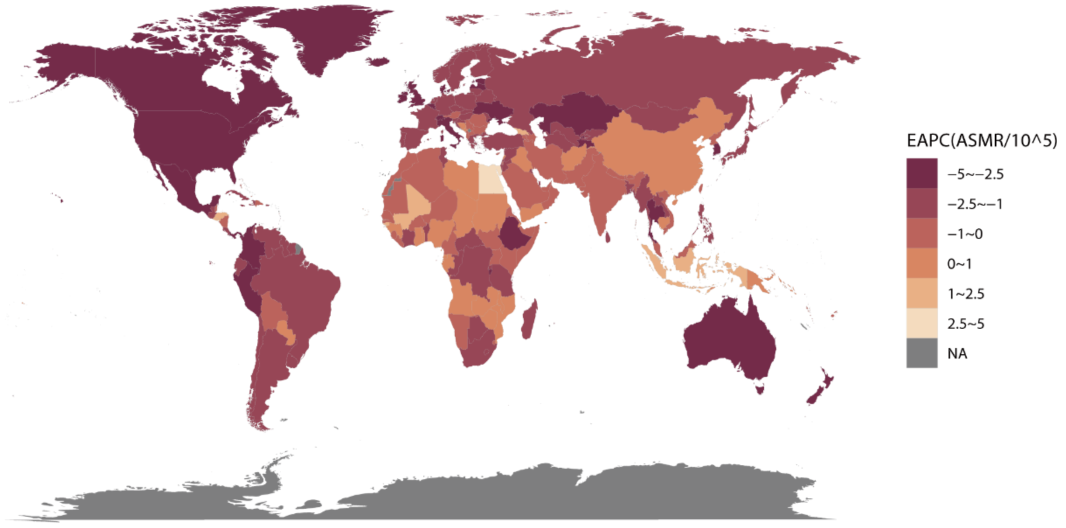

B

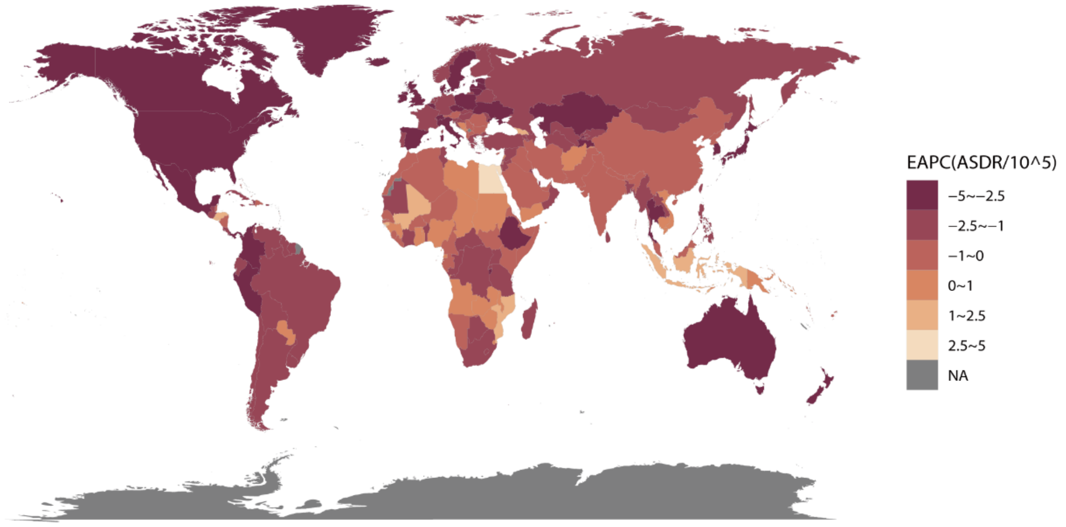

Fig. S1. The global distribution of EAPC in ASMR (A) and ASDR (B) of lung cancer attributable to secondhand smoke from 1990 to 2021. ASMR, age-standardized mortality rate; ASDR, age-standardized DALYs rate; DALYs, disability-adjusted life-years; EAPC, estimated annual percentage change.

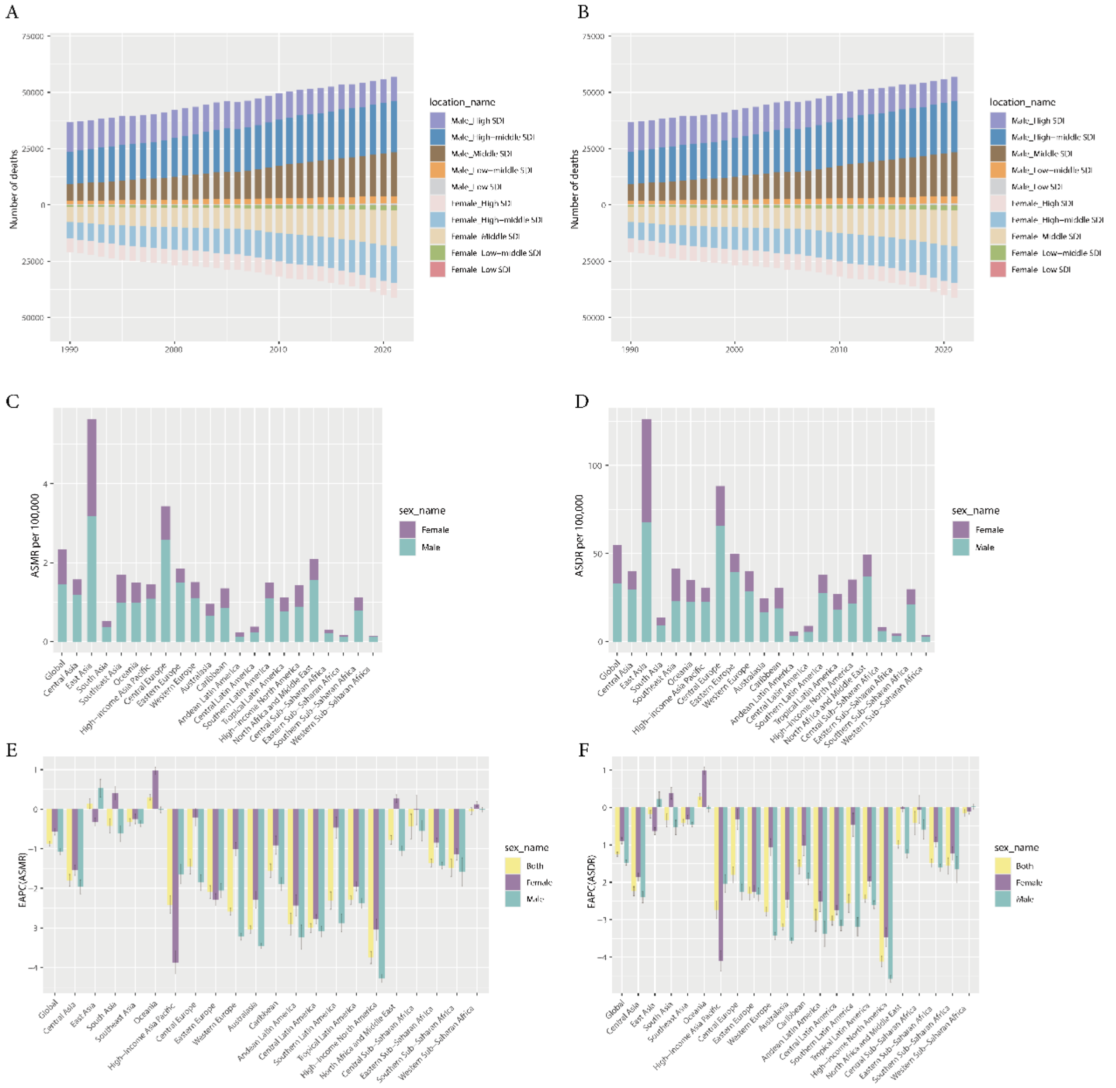

Fig. S2. Spatiotemporal distribution of lung cancer attributable to secondhand smoke. The global burden of lung cancer deaths (A) and DALYs (B) attributable to secondhand smoke from 1990 to 2021 by sex and SDI region. ASMR (C) and ASDR (D) of lung cancer attributable to secondhand smoke in 2021, by sex and region. EAPC of ASMR (E) and ASDR (F) for lung cancer attributable to secondhand smoke from 1990 to 2021 by sex across global and 21 regional locations (error bars represent 95% confidence intervals of the EAPC). ASMR, age-standardized mortality rate; ASDR, age-standardized DALYs rate; EAPC, estimated annual percentage change; SDI, socio-demographic index.

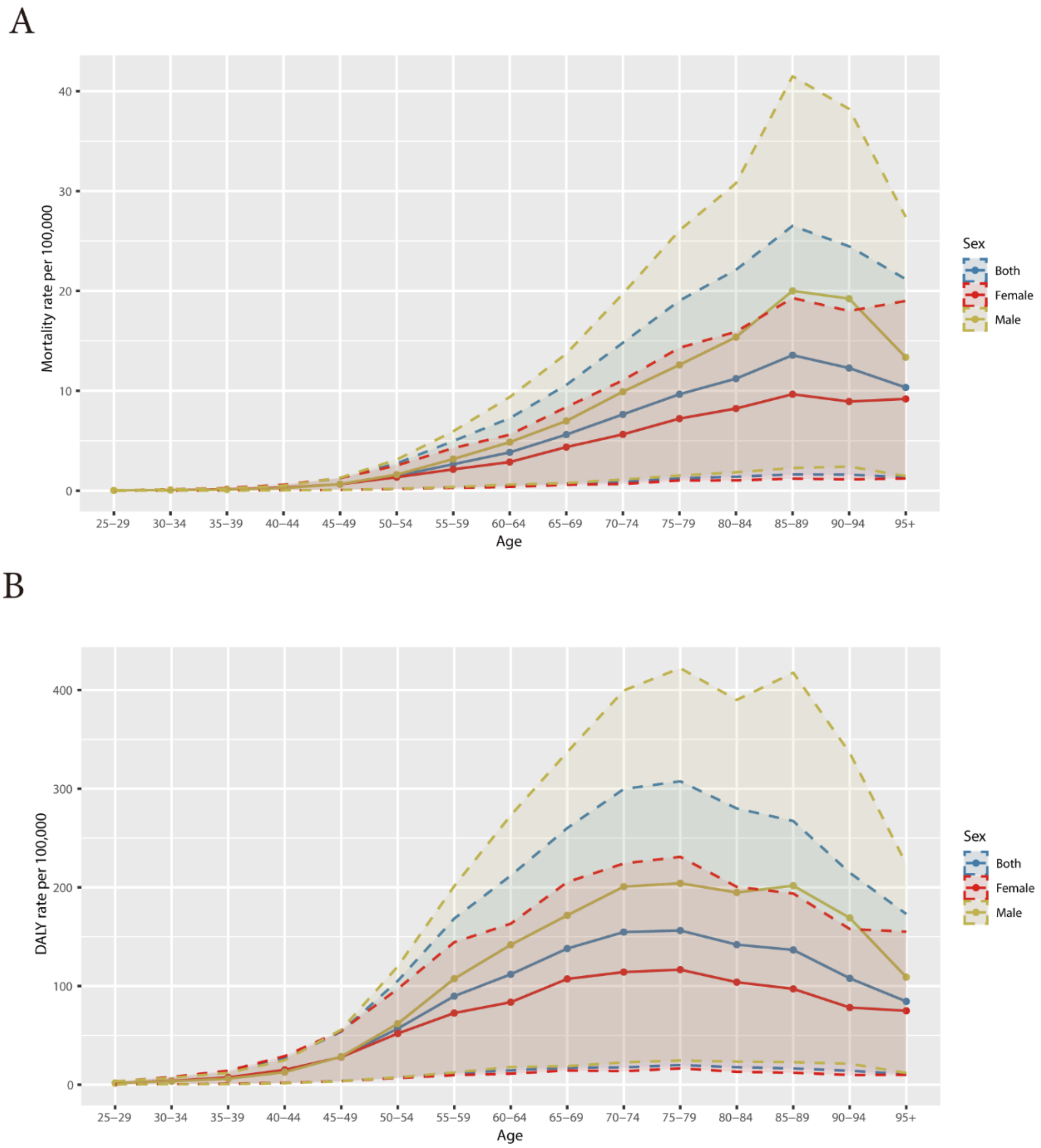

Fig. S3. Age-specific rates of global deaths (A) and DALYs (B) of lung cancer attributable to secondhand smoke with 95% UI, by sex, in 2021. DALYs, disability-adjusted life-years; UI, uncertainty interval.

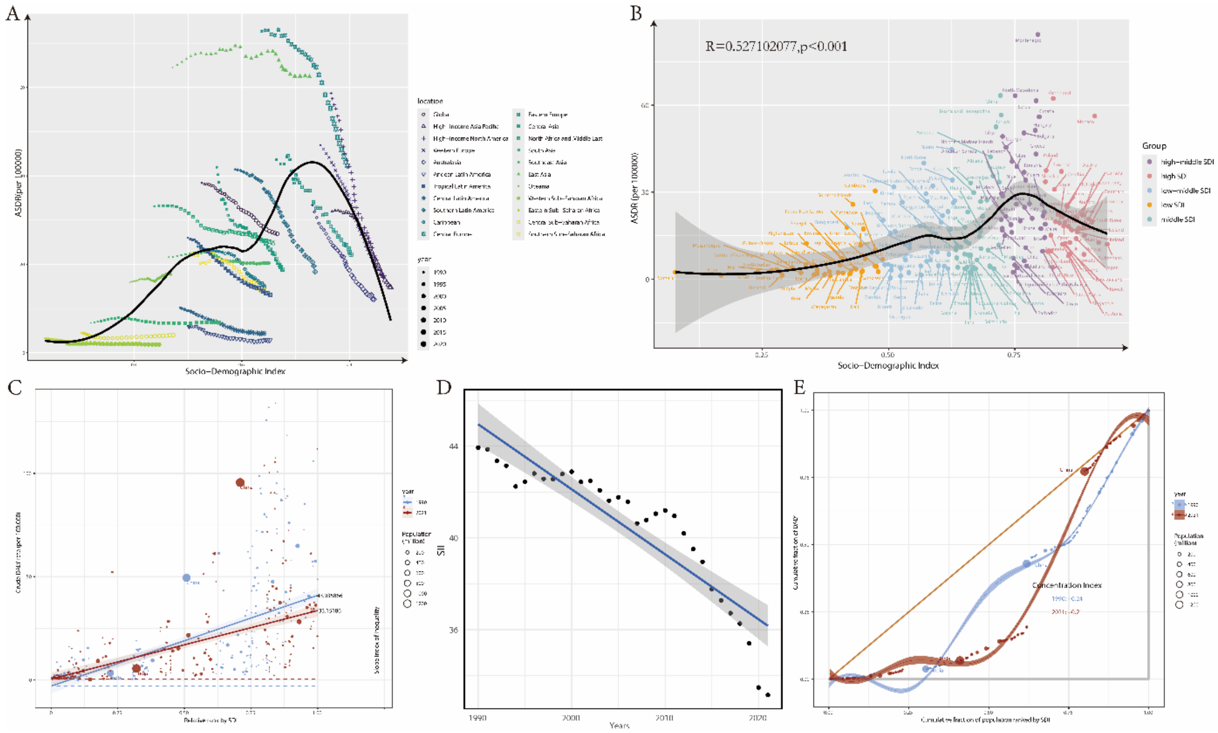

Fig. S4. Correlations between ASDR of lung cancer attributable to secondhand smoke and SDI. (A) ASDR of lung cancer attributable to secondhand smoke at the global level and 21 regions, by SDI, from 1990 to 2021. (B) ASDR of lung cancer attributable to secondhand smoke in 204 countries and territories, by SDI, in 2021. Black line represents the expected ASMR and ASDR based on SDIs in all locations. (C) Absolute income-related healthy inequality in lung cancer attributable to secondhand smoke burden, presented using regression lines, 1990 vs. 2021. (D) Trendline demonstrates the trend in SII from 1990 to 2021. (E) Relative income- related healthy inequality in lung cancer attributable to secondhand smoke burden, presented using concentration curves, 1990 vs. 2021. ASDR, age-standardized DALYs rate; SDI, socio-demographic index.

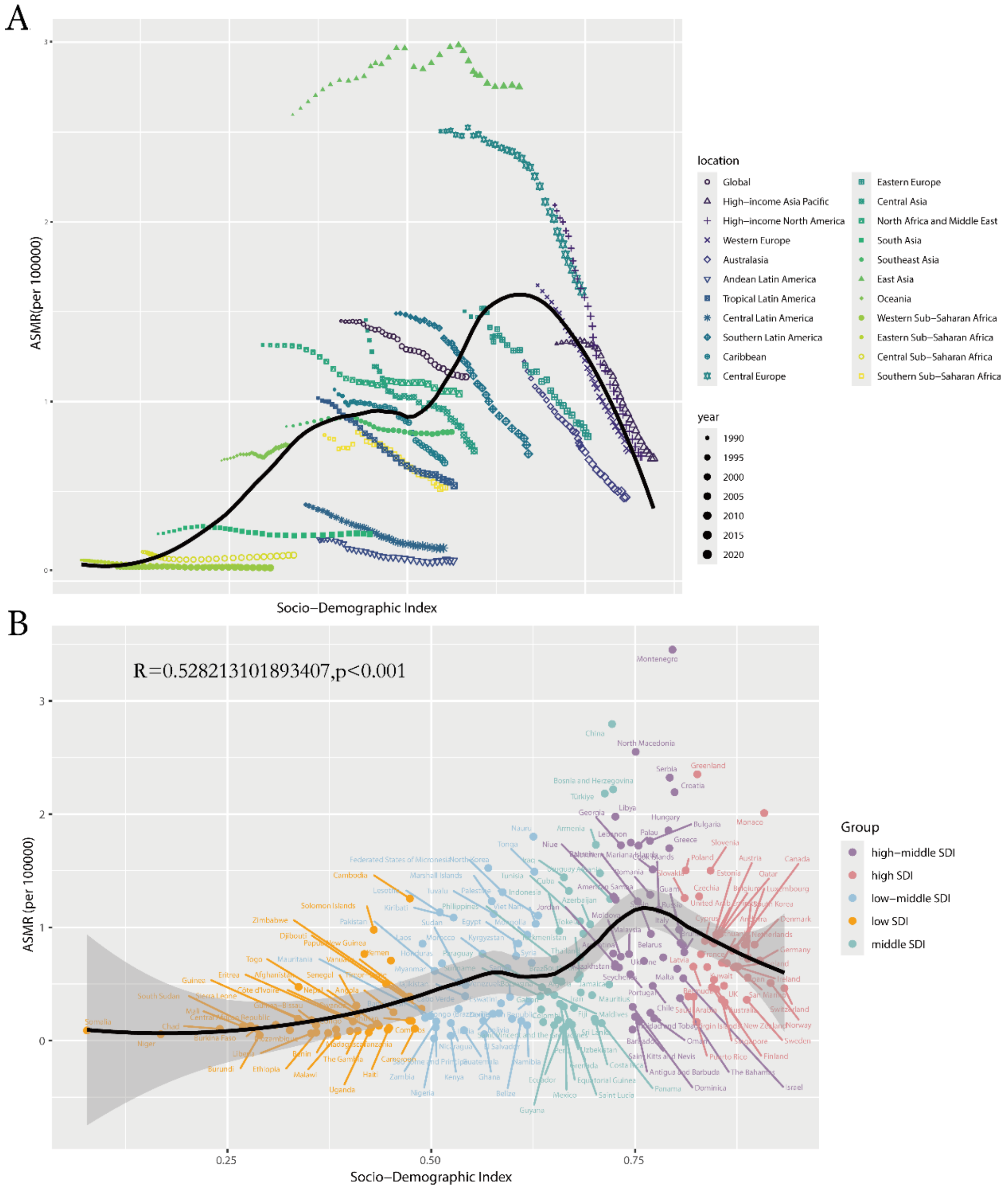

Fig. S5. Correlations between ASMR of lung cancer attributable to secondhand smoke and SDI at the regional (A) and national (B) level. ASMR of lung cancer attributable to secondhand smoke at the global level and 21 regions, by SDI, from 1990 to 2021 (A). ASMR of lung cancer attributable to secondhand smoke in 204 countries and territories, by SDI, in 2021 (B). Black line represents the expected ASMR and ASDR based on SDIs in all locations;

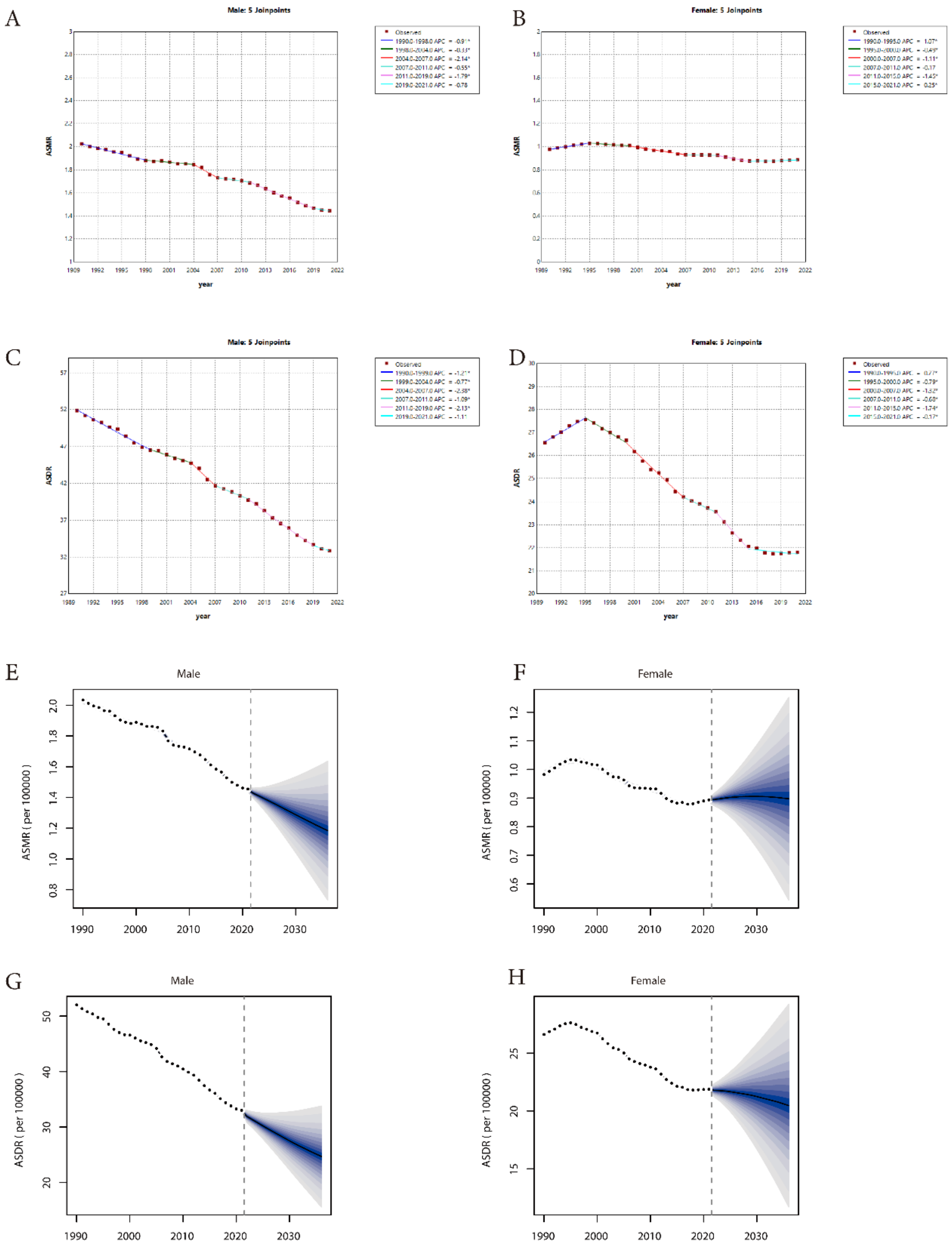

Fig. S6. Joinpoint regression analysis from 1990 to 2021 and the projected trends from 2021 to 2036. (A) ASMR for males. (B) ASMR for females. (C) ASDR for males. (D) ASDR for females. (E) Predicted ASMR for males. (F) Predicted ASMR for females. (G) Predicted ASDR for males. (H) Predicted ASDR for females. ASMR, age-standardized mortality rate; ASDR, age-standardized DALYs rate; DALYs, disability-adjusted life-years.

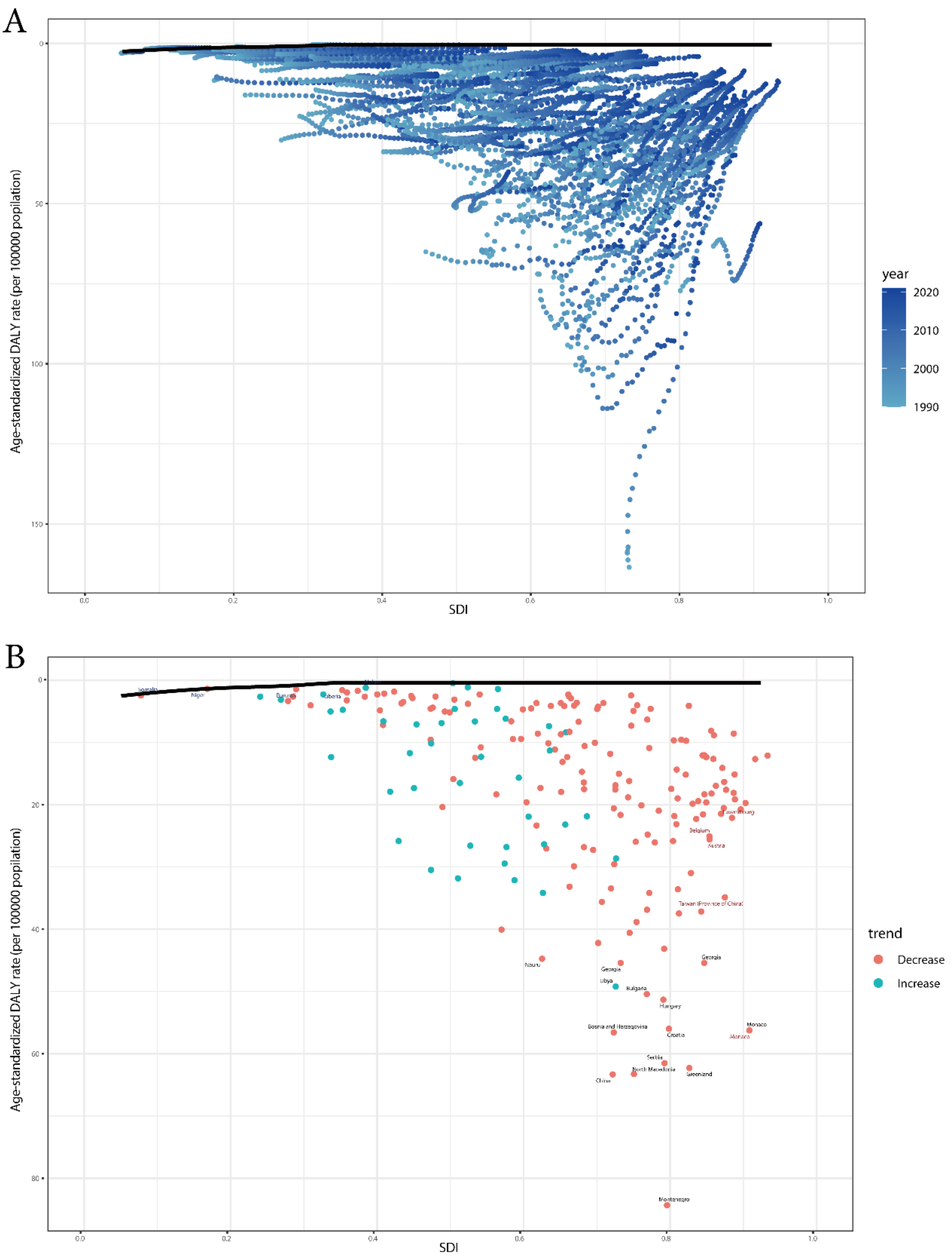

Fig. S7. Frontier analysis based on SDI and age-standardized DALY rate of lung cancer attributable to secondhand smoke in 2021. The frontier is delineated in solid black color; countries and territories are represented as dots. The top 15 countries with the largest effective difference (largest lung cancer attributable to secondhand smoke DALYs gap from the frontier) are labeled in black; examples of frontier countries with low SDI (< 0.5) and low effective difference are labeled in black blue (e.g., Somalia, Niger, Burundi, Liberia), and examples of countries and territories with high SDI (> 0.85) and relatively high effective difference for their level of development are labeled in red (e.g., Luxembourg, Belgium, Austria, Monaco. Red dots

indicate an increase in age-standardized lung cancer attributable to secondhand smoke DALYs rate from 1990 to 2021; blue dots indicate a decrease in age-standardized lung cancer attributable to secondhand smoke DALYs rate between 1990 and 2021. SDI: Socio-demographic index; DALYs: Disability-Adjusted Life Years.

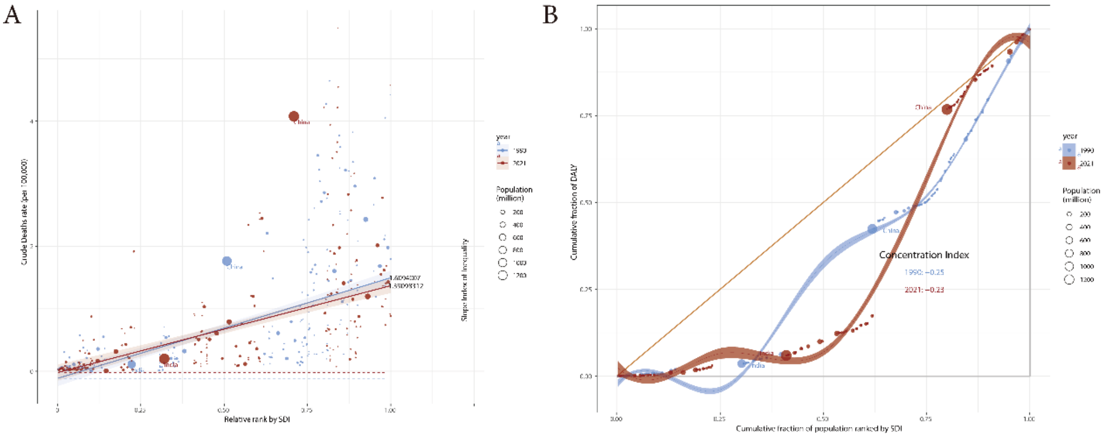

Fig. S8. SII analysis. (A) Absolute income-related healthy inequality in lung cancer attributable to secondhand smoke burden, presented using regression lines, 1990 vs. 2021. Concentration index analysis. (B) Relative income- related healthy inequality in lung cancer attributable to secondhand smoke burden, presented using concentration curves, 1990 vs. 2021.

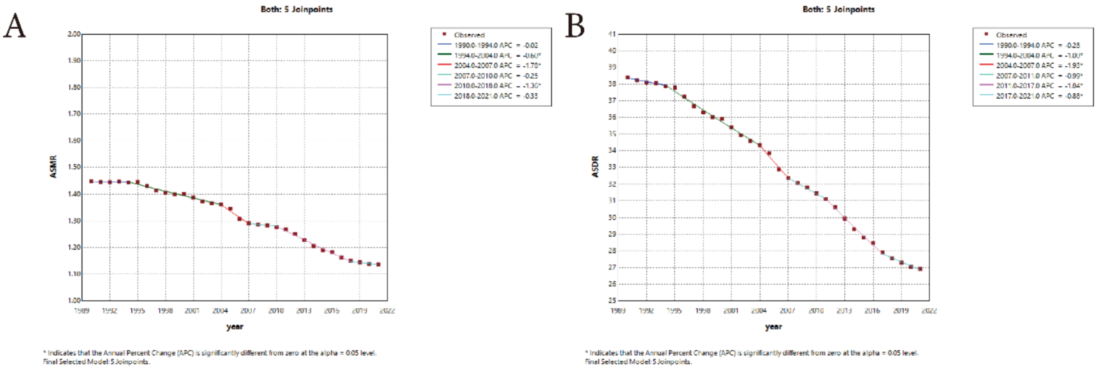

Fig. S9. Joinpoint regression analysis of the sex-specific ASMR and ASDR for lung cancer attributable to secondhand smoke from 1990 to 2021. (A) ASMR for both. (B) ASDR for both. ASMR, age-standardized mortality rate; ASDR, age-standardized DALYs rate; DALYs, disability-adjusted life-years.

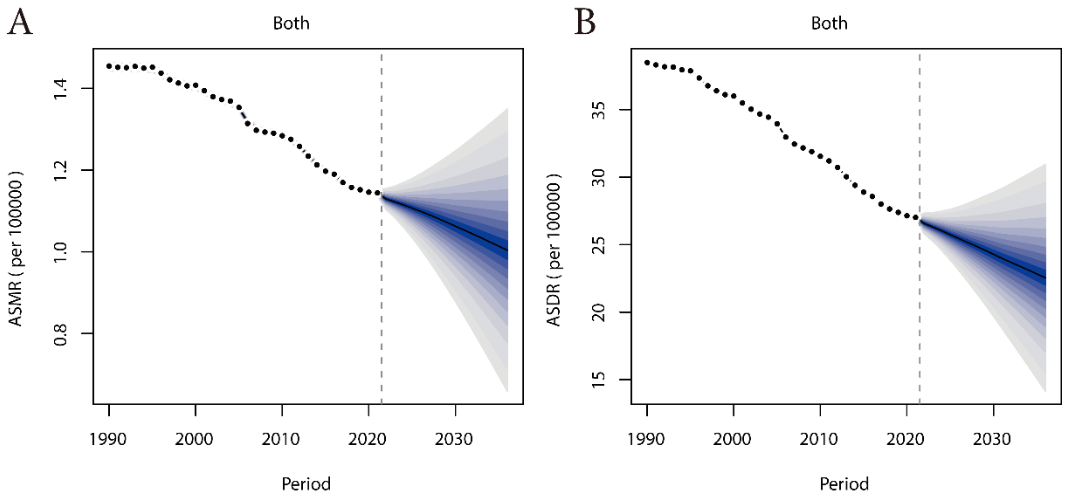

Fig. S10. ASMR and ASDR BAPC predictive analysis for lung cancer attributable to secondhand smoke from 1990 to 2021. (A) Predicted ASMR for both. (B) Predicted ASDR for both. ASMR, age-standardized mortality rate; ASDR, age-standardized DALYs rate; DALYs, disability-adjusted life-years.

© 2025 Yu Y. et al.
